# GVHD occurrence does not reduce AML relapse following PTCy-based haploidentical transplantation: a study from the ALWP of the EBMT

**DOI:** 10.1186/s13045-023-01403-x

**Published:** 2023-02-13

**Authors:** Frédéric Baron, Myriam Labopin, Johanna Tischer, Anna Maria Raiola, Jan Vydra, Didier Blaise, Patrizia Chiusolo, Friedrich Stölzel, Renato Fanin, Patrice Chevallier, Arnon Nagler, Fabio Ciceri, Mohamad Mohty

**Affiliations:** 1 Laboratory of Hematology, GIGA-I3, University of Liege and CHU of Liège, Sart-Tilman, 4000 Liège, Belgium; 2 EBMT Paris Study Office/CEREST-TC, Paris, France; 3 Department of Hematology, Saint Antoine Hospital, Paris, France; 4 INSERM UMR 938, Paris, France; 5 Sorbonne University, Paris, France; 6 Department of Internal Medicine III, LMU, University Hospital of Munich, Campus Grosshadern, Munich, Germany; 7 IRCCS Ospedale Policlinico San Martino, Genoa, Italy; 8 Institute of Hematology and Blood Transfusion, Prague, Czech Republic; 9 Programme de Transplantation et d’immunothérapie Cellulaire, Management Sport Cancer Lab, Institut Paoli Calmettes, Aix Marseille University, Marseille, France; 10 Dipartimento di Diagnostica per Immagini, Radioterapia Oncologica ed Ematologia, Fondazione Policlinico Universitario A. Gemelli IRCCS, Rome, Italy; 11 University Hospital Dresden, Medizinische Klinik und Poliklinik, TU Dresden, Dresden, Germany; 12 University Hospital Schleswig-Holstein, Kiel, Germany; 13 Division of Hematology, Azienda Ospedaliero Universitaria di Udine, Udine, Italy; 14 Dept. D`Hematologie, CHU Nantes, Nantes, France; 15 Division of Hematology and Bone Marrow Transplantation, The Chaim Sheba Medical Center, Tel-Hashomer, Ramat-Gan, Israel; 16 Haematology and BMT, Ospedale San Raffaele S.R.L., Milan, Italy

**Keywords:** AML, Acute myeloid leukemia, HLA-haploidentical, Mismatched unrelated donor, Post-transplant cyclophosphamide, PTCy

## Abstract

The association between graft-versus-host disease (GVHD) occurrence and acute myeloid leukemia (AML) relapse in patients treated with HLA-haploidentical allogeneic hematopoietic stem cell transplantation (Haplo-HCT) with post-transplant cyclophosphamide (PTCy)-based GVHD prophylaxis has remained debated. Here, we addressed this issue in patients with active AML at transplantation. 2-year cumulative incidences of relapse and leukemia-free survival (LFS) were 49% and 32.3%, respectively. There were no associations between acute nor chronic GVHD of any grade and lower relapse incidence. However, grade I acute GVHD was associated with better LFS (HR = 0.71, 95% CI 0.51–0.99, *P* = 0.04). In contrast, grade III–IV acute (HR = 3.09, 95% CI 1.87–5.12, *P* < 0.0001) as well as extensive chronic (HR = 3.3, 95% CI 1.81–6.04, *P* = 0.0001) GVHD correlated with higher nonrelapse mortality leading to lower LFS (HR = 1.36, 95% CI 0.99–1.86, *P* = 0.056 and HR = 1.97, 95% CI 1.35–2.89, *P* = 0.0004, respectively). In conclusion, these data suggest a dissociation of graft-versus-leukemia effects from GVHD in patients with active AML treated with PTCy-based Haplo-HCT.

**Supplementary Information:**

The online version contains supplementary material available at 10.1186/s13045-023-01403-x.

To the Editor

Allogeneic hematopoietic stem cell transplantation (allo-HCT) has remained the best option for patients with relapsed/refractory acute myeloid leukemia (AML) [1]. This approach relies on graft-versus-leukemia (GVL) effects for leukemia eradication. In patients receiving grafts from HLA-matched donors, numerous studies have demonstrated a tight association between occurrence of graft-versus-host disease (GVHD) and lower risk of relapse [2–4].

Post-transplant cyclophosphamide (PTCy)-based GVHD prophylaxis has revolutionized the field of human leukocyte antigen (HLA)-haploidentical hematopoietic cell transplantation (Haplo-HCT) [5, 6]. Consequently, Haplo-HCT is nowadays frequently used as treatment for relapsed/refractory AML patients [7]. A recent systems biology analysis in patients with PTCy-based GVHD prophylaxis demonstrated different signatures associated with GVHD and GvL effects [8]. In addition, another study observed different T-cell phenotypes associated with GVHD or GvL in PTCy-Allo-HCT recipients [9]. These observations prompted us to perform a large retrospective study in the EBMT registry aimed at assessing whether PTCy given in the Haplo-HCT setting might dissociate GVL effects from GVHD in patients with active AML at transplantation, a subgroup of patients who particularly rely on GVL effects for leukemic cell eradication. Population selection criteria included ≥ 18 years of age at transplantation, Haplo-HCT between 2010 and 2020 with PTCy, no prior allo-HCT, and primary refractory or relapsed AML (i.e. all patients had active disease at the time of transplant conditioning initiation).

The analyses were carried out in a total of 670 patients (Additional file 1: Table 1). The 180-day incidences of grade II-IV and grade III-IV acute GVHD were 30.8% (95% CI 27.4–34.3%) and 13.3% (95% CI 10.9–16%), respectively. These incidences were 21% and 8%, respectively, in BM recipients versus 35% (*P* = 0.001) and 16% (*P* = 0.008), respectively, in PBSC recipients. The 2-year cumulative incidences of chronic and extensive chronic GVHD were 26.8% (95% CI 23.4–30.3%) and 13% (95% CI 10.5–15.8%), respectively. There was no impact of stem cell source on chronic GVHD incidence. However, in vivo T-cell depletion was associated with a lower incidence of chronic GVHD (17% versus 28%, *P* = 0.027).

**Table 1:** Final model stratified on landmark at time intervals from day of allo-HCT to day + 360 by 30 days

	RI		NRM		LFS		OS	
	HR	*P*	HR	*P*	HR	*P*	HR	*P*
No acute GVHD (*n* = 320, ref.)	1		1		1		1	
Acute GVHD I (*n* = 144)	0.82 (0.56–1.21)	0.32	0.53 (0.27–1.01)	0.055	0.71 (0.51–0.99)	0.042	0.68 (0.48–0.97)	0.032
Acute GVHD II (= 117)	0.83 (0.54–1.26)	0.38	0.78 (0.4–1.52)	0.46	0.8 (0.56–1.15)	0.23	0.86 (0.6–1.24)	0.42
Acute GVHD III–IV (*n* = 89)	0.87 (0.55–1.36)	0.54	3.09 (1.87–5.12)	< 0.0001	1.36 (0.99–1.86)	0.056	1.32 (0.88–1.97)	0.17
No cGVHD (reference)	1		1		1		1	
Limited cGVHD	0.8 (0.43–1.49)	0.48	1.23 (0.54–2.81)	0.63	0.93 (0.57–1.52)	0.77	0.84 (0.48–1.49)	0.56
Extensive cGVHD	1.34 (0.74–2.42)	0.33	3.3 (1.81–6.04)	0.0001	1.97 (1.35–2.89)	0.0004	1.95 (1.29–2.94)	0.001
Age (per 10 y)*	0.9 (0.82–0.99)	0.038	1.56 (1.27–1.92)	< 0.0001	1.03 (0.94–1.13)	0.48	1.07 (0.98–1.18)	0.14
Sec. AML*	0.79 (0.54–1.15)	0.22	0.87 (0.52–1.46)	0.61	0.85 (0.63–1.15)	0.3	0.94 (0.69–1.28)	0.7
Adverse cytogenetics*	1.83 (1.37–2.45)	< 0.0001	1.33 (0.86–2.06)	0.2	1.68 (1.32–2.13)	< 0.0001	1.65 (1.28–2.12)	0.0001
Year of HCT*	0.97 (0.92–1.03)	0.3	0.97 (0.88–1.07)	0.55	0.97 (0.93–1.02)	0.27	0.98 (0.93–1.03)	0.42
KPS90*	0.88 (0.67–1.16)	0.37	0.61 (0.4–0.93)	0.022	0.82 (0.65–1.02)	0.08	0.82 (0.64–1.04)	0.11
Female to male*	0.8 (0.56–1.15)	0.23	1.43 (0.9–2.28)	0.13	0.97 (0.74–1.26)	0.8	1.08 (0.81–1.44)	0.58
Patient CMV positive*	1.19 (0.84–1.68)	0.32	1.01 (0.63–1.64)	0.96	1.12 (0.85–1.48)	0.42	1.11 (0.83–1.47)	0.49
Donor CMV positive*	1.14 (0.85–1.53)	0.37	0.73 (0.47–1.14)	0.17	1 (0.78–1.27)	0.97	1.02 (0.79–1.33)	0.86
PB vs BM*	0.94 (0.69–1.28)	0.72	1.66 (1.01–2.72)	0.046	1.07 (0.83–1.39)	0.58	1.1 (0.84–1.45)	0.47
RIC vs MAC*	1.11 (0.83–1.5)	0.48	0.88 (0.52–1.46)	0.61	1.06 (0.82–1.38)	0.64	1.15 (0.87–1.52)	0.33
In vivo TCD*	1.59 (1.03–2.44)	0.035	0.89 (0.4–1.97)	0.78	1.34 (0.9–1.99)	0.15	1.28 (0.86–1.92)	0.22

*Co-variates in the multivariate models; *Ref.* Reference; *RI* incidence of relapse; *NRM* nonrelapse mortality; *LFS* leukemia-free survival; *OS* overall survival; *GVHD* graft-versus-host disease; *cGVHD* chronic GVHD; *HCT* hematopoietic cell transplantation; *CMV* cytomegalovirus; *PB* peripheral blood stem cells; *BM* bone marrow; *RIC* reduced-intensity conditioning; *MAC* myeloablative conditioning; in vivo* TCT* in vivo T-cell depletion. There was no interaction between stem cell source (PB vs. BM) and the impact of GVHD on transplantation outcome

The impact of GVHD on transplantation outcomes was assessed using dynamic landmarking *i.e.* a method including a series of landmark analyzes at each time interval of 30 days from allo-HCT to day 365 (Table 1, see Additional file 1 for more details) [10].

There was no impact of acute nor of chronic GVHD on relapse incidence (Table 1 and Fig. 1). There were no associations between either grade II acute GVHD nor limited chronic GVHD on NRM, LFS nor OS in dynamic landmarking models (Table 1). However, grade III-IV acute GVHD was associated with higher NRM (HR = 3.09, 95% CI 1.87–5.12, *P* < 0.0001) and a statistical trend for lower LFS (HR = 1.36, 95% CI 0.99–1.86, *P* = 0.056) (Fig. 1). In contrast, grade I acute GVHD was associated with a trend for lower NRM (HR = 0.53, 95% CI 0.27–1.01, *P* = 0.055) and better LFS (HR = 0.71, 95% CI 0.51–0.99, *P* = 0.042) and OS (HR = 0.68, 95% CI 0.48–0.97, *P* = 0.032). We do not have a biological explanation for the lower NRM in patients with grade 1 acute GVHD. Future studies needed to evaluate whether this is due to a better immune reconstitution in patients with grade I acute GVHD. Finally, extensive chronic GVHD was associated with higher NRM (HR = 3.3, 95% CI 1.81–6.04, *P* < 0.0001) and lower LFS (HR = 1.97, 95% CI 1.35–2.89, *P* = 0.0004) and OS (HR = 1. 95, 95% CI 1.29–2.94, *P* = 0.001) (Fig. 1).

**Fig. 1:**
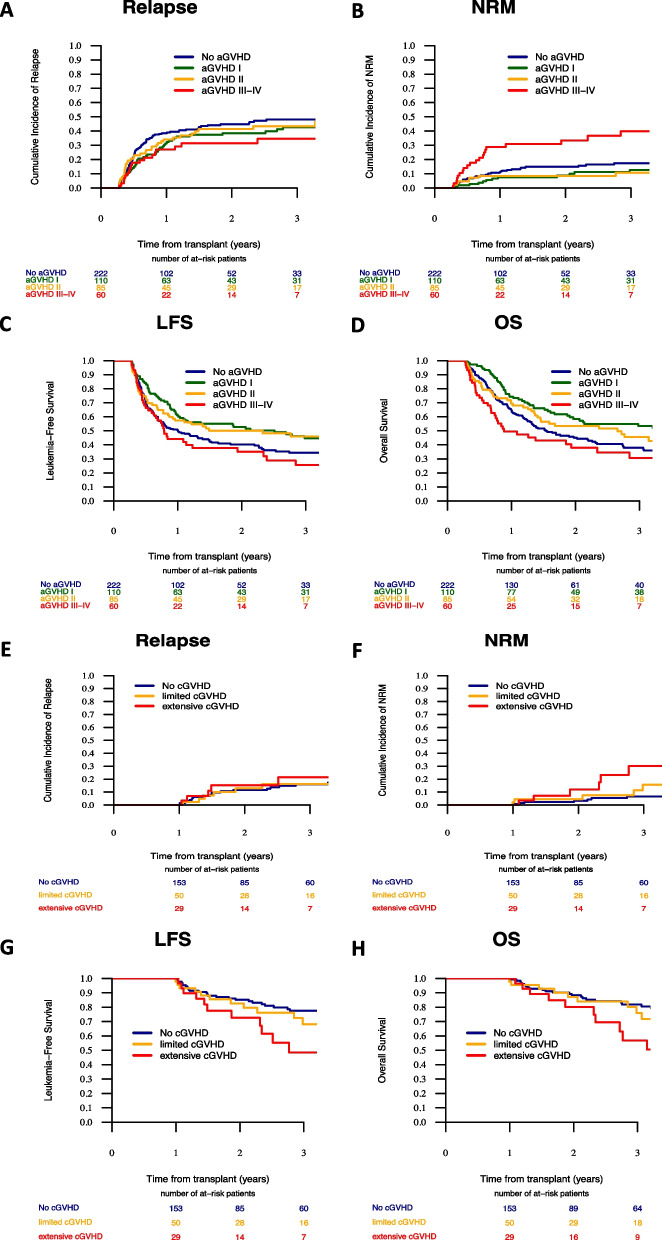
**A**–**D** Day-100 landmark analyses (*n* = 477) showing the impact of grade I, II and grade III–IV acute GVHD on: **A** relapse incidence (*P* = 0.39); **B** incidence of nonrelapse mortality (NRM, *P* = 0.001); **C** Leukemia-free survival (LFS, *P* = 0.005); **D** overall survival (OS, *P* = 0.002). **E**–**H** Day-365 landmark analyses (*n* = 234) showing the impact of limited and extensive chronic GVHD on: **E** relapse incidence (*P* = 0.8); **F** NRM (*P* = 0.021); **G** LFS (*P* = 0.11); **H** OS (*P* = 0.014)

Our results differ from what has been observed by the Baltimore group in patients receiving Haplo-HCT with PTCy-based GVHD prophylaxis after nonmyeloablative conditioning as treatment of various hematological malignancies (*n* = 340) [11]. Indeed, in that study, grade II acute GVHD was associated with a lower risk of relapse. Our observations are, however, concordant with recent observations in another large population of patients treated with Haplo-HCT as treatment for AML in CR (*n* = 805) [10] and with data in humanized mouse models of GVHD in which it was demonstrated that PTCy attenuated GVHD without abrogating graft-versus-leukemia effects [12].

The absence of association between GVHD occurrence and the risk of relapse might suggest that in vivo T-cell depletion could be particularly suitable in the Haplo-HCT PTCy setting. However, we observed that ATG was associated with higher relapse incidence in multivariate analysis, without significantly affecting OS and LFS.

In conclusion, we demonstrated in a cohort of patients with active AML at transplantation treated with PTCy-based T-cell repleted Haplo-HCT that occurrence of GVHD did not decrease the risk of relapse suggesting a dissociation of GvL effects from GVHD in this transplantation setting.

## Supplementary Information

**Additional file 1**. Supplemental data.

## Data Availability

ML and MM had full access to all the data in the study. Data are available upon reasonable request. Please contact Dr Myriam Labopin (myriam.labopin@upmc.fr).

## Abbreviations

Allo-HCT: Allogeneic hematopoietic stem cell transplantation
AML: Acute myeloid leukemia
ATG: Anti-thymocyte globulin
GVHD: Graft-versus-host disease
GVL: Graft-versus-leukemia effect
Haplo-HCT: HLA-haploidentical stem cell transplantation
LFS: Leukemia-free survival
NRM: Nonrelapse mortality
OS: Overall survival
PTCy: Post-transplant cyclophosphamide

## References

[1] Zhang X-H, Chen J, Han M-Z, Huang H, Jiang E-L, Jiang M (2021). The consensus from The Chinese Society of Hematology on indications, conditioning regimens and donor selection for allogeneic hematopoietic stem cell transplantation: 2021 update. J Hematol Oncol.

[2] Weiden PL, Sullivan KM, Flournoy N, Storb R, Thomas ED (1981). Antileukemic effect of chronic graft-versus-host disease: contribution to improved survival after allogeneic marrow transplantation. N Engl J Med.

[3] Horowitz MM, Gale RP, Sondel PM, Goldman JM, Kersey J, Kolb HJ (1990). Graft-versus-leukemia reactions after bone marrow transplantation. Blood.

[4] Baron F, Labopin M, Niederwieser D, Vigouroux S, Cornelissen JJ, Malm C (2012). Impact of graft-versus-host disease after reduced-intensity conditioning allogeneic stem cell transplantation for acute myeloid leukemia: a report from the Acute Leukemia Working Party of the European group for blood and marrow transplantation. Leukemia.

[5] Kanakry CG, Fuchs EJ, Luznik L (2016). Modern approaches to HLA-haploidentical blood or marrow transplantation. Nat Rev Clin Oncol.

[6] Sanz J, Galimard J-E, Labopin M, Afanasyev B, Angelucci E, Ciceri F (2020). Post-transplant cyclophosphamide after matched sibling, unrelated and haploidentical donor transplants in patients with acute myeloid leukemia: a comparative study of the ALWP EBMT. J Hematol Oncol.

[7] Baron F, Labopin M, Tischer J, Ciceri F, Raiola AM, Blaise D (2022). Human leukocyte antigen-haploidentical transplantation for relapsed/refractory acute myeloid leukemia: Better leukemia-free survival with bone marrow than with peripheral blood stem cells in patients ≥55 years of age. Am J Hematol.

[8] McCurdy SR, Radojcic V, Tsai H-L, Vulic A, Thompson E, Ivcevic S (2022). Signatures of GVHD and relapse after post-transplant cyclophosphamide revealed by immune profiling and machine learning. Blood.

[9] Zhao C, Bartock M, Jia B, Shah N, Claxton DF, Wirk B (2022). Post-transplant cyclophosphamide alters immune signatures and leads to impaired T cell reconstitution in allogeneic hematopoietic stem cell transplant. J Hematol Oncol.

[10] Shimoni A, Labopin M, Angelucci E, Blaise D, Ciceri F, Koc Y (2022). The association of graft-versus-leukemia effect and graft-versus host disease in haploidentical transplantation with post-transplant cyclophosphamide for AML. Bone Marrow Transplant.

[11] McCurdy SR, Kanakry CG, Tsai H-L, Kasamon YL, Showel MM, Bolaños-Meade J (2018). Grade II acute graft-versus-host disease and higher nucleated cell graft dose improve progression-free survival after HLA-haploidentical transplant with post-transplant cyclophosphamide. Biol Blood Marrow Transplant.

[12] Ritacco C, Cem Köse M, Courtois J, Canti L, Beguin C, Dubois S, et al. Post-transplant cyclophosphamide prevents xenogeneic graft-versus-host disease while depleting proliferating regulatory T cells. iScience. 2023; in press. 10.1016/j.isci.2023.106085 PMC9947306 36843851

