# Supplementary material for: GVHD occurrence does not reduce AML relapse following PTCy-based haploidentical transplantation: a study from the ALWP of the EBMT
Source: J Hematol Oncol. 2023 Feb 13;16:10. doi: 10.1186/s13045-023-01403-x (PMC9923893; doi:10.1186/s13045-023-01403-x)
Supplement: Supplementary file 1 — Additional file 1. Supplemental data. [file 13045_2023_1403_MOESM1_ESM.docx]

**Supplemental material and methods**

**Definitions**

Reduced intensity conditioning (RIC) was defined as regimens combining fludarabine with either <6 Gy total body irradiation (TBI), ≤8mg/kg busulfan, or ≤140 mg/m^2^ melphalan or with other nonmyeloablative drugs as previously reported^1^ ^2^. Acute and chronic GVHD were graded according to previously reported criteria^3^. Haplo-HCT was defined as allo-HCT from two or more HLA-mismatched related donors. Comorbidities at transplantation were determined using the hematopoietic cell transplantation-specific comorbidity-index (HCT-CI) score^4^. Cytogenetic risk was stratified according to the MRC-UK classification, as previously reported^5^ ^6^.

**Supplemental statistical analyses**

Outcome studied were relapse incidence (defined as the presence of 5% BM blasts and/or reappearance of the underlying disease after transplantation), non-relapse mortality (NRM), leukemia-free survival (LFS), and overall Survival (OS). OS was defined as the time from allo-HCT to death, regardless of the cause. LFS was defined as survival with no evidence of relapse or progression. NRM was defined as death without evidence of relapse or progression. Cumulative incidence was used to estimate the endpoints of acute and chronic GVHD, NRM and RI to accommodate for competing risks. To study acute and chronic GVHD, we considered relapse and death to be competing events. Probabilities of OS, LFS and GRFS were calculated using the Kaplan–Meier method. Univariate analyses were done using the Gray’s test for cumulative incidence functions and the log rank test for OS and LFS.

The primary endpoint for assessing the impact of acute and chronic GVHD on the outcome was RI. Secondary endpoints were NRM, LFS and OS. For this purpose, we used a series of landmark analyses ^7^ ^8^. From the original data set we constructed data sets for 12 landmark time points from Haplo-HCT (day 0) to 360 days by 30 days, selecting patients alive in remission at these time points. All potential risk factors were included as covariates in the multivariate model. In order to take into account for the “overlap” between landmark data sets, and since the data of the same patient are used repeatedly in the different landmark strata, we used a stacked data set containing all the landmark data sets and the final model was stratified on the landmark and standard errors obtained by taking into account the “clustering” of the data using the sandwich estimators of Lin and Wei (1989) ^9^. We also fitted a simple Cox model at day 30, 100, 180 and 360. Hazard ratios (HR) and 95% confidence intervals (95% CI) are reported.

Statistical analyses were performed with 4.1.1 (R Core Team (2021). R: A language and environment for statistical computing. R Foundation for Statistical Computing, Vienna, Austria. URL <https://www.R-project.org/>.), packages ‘survival’, ‘cmprsk’ and ‘dynpred’.

**Supplemental Results**

**Patients**

A total of 803 patients met the population selection criteria. One hundred and thirty-three patients were excluded from the analyses because they died before achieving engraftment (n=90), received a second transplantation for graft failure (n=10), because of missing data in the registry on GVHD (n=31), or missing information about conditioning intensity (n=2). Thus, the analyses were carried out in a total of 670 patients. The number of patients at risk (i.e. alive without relapse) at the beginning of the landmark interval were 645 at 30 days, 477 at 100 days, 343 at 180 days, and 232 at 360 days. Briefly, median patient age was 55.5 years (IQR, 42.8-68.3 years). Status at transplantation was primary refractory (n=366, 55%), first relapse (n=238, 35%) or second or more advanced relapse (n=66, 10%).

Conditioning was myeloablative (MAC) in 289 patients and RIC in the remaining 381 patients. Stem cell source was bone marrow (BM) in 216 patients (32%) and peripheral blood stem cells (PBSC) in the 454 (68%) remaining patients. Eighty-five patients (13%) were given *in vivo* T-cell depletion.

**Transplantation outcomes**

The 2-year cumulative incidences of relapse and NRM were 49% (95% CI: 45-52.8%) and 18.7% (95% CI: 15.8-21.9%), respectively. The 2-year LFS and OS were 32.3% (95% CI: 28.6-36.1%) and 36.1% (95% CI: 32.2-40%), respectively.

**Landmark analyses**

Factors associated with the relapse incidence in the final model stratified on landmark time points included adverse cytogenetics (HR=1.83, 95% CI: 1.37-2.45, P<0.0001) and *in vivo* T-cell depletion (HR=1.59, 95% CI 1.03-2.44, P=0.032). Besides grade III-IV acute and extensive chronic GVHD, older patient age (HR=1.56, 95%CI: 1.27-1.91, P<0.0001) and PBSC as stem cell source (HR=1.66, 95%CI: 1.01-2.72, P=0.046) were also associated with higher NRM without significantly affecting LFS and OS. In contrast, Karnofsky performance score >=90 was associated with lower NRM (HR=0.61, 95%CI: 0.40-0.93, P=0.022). Finally, adverse-risk cytogenetics correlated with lower LFS (HR=1.68, 95%CI: 1.32-2.13, P<0.0001) and OS (HR=1.65, 95%CI: 1.28-2.12, P=0.0001).

In addition, it should be stressed that the absence of association between GVHD occurrence and the risk of relapse was also observed when ATG patients were removed from the analyses (supplemental Table 2).

**References**

1 Baron F, Ruggeri A, Beohou E, Labopin M, Sanz G, Milpied N *et al.* RIC versus MAC UCBT in adults with AML: A report from Eurocord, the ALWP and the CTIWP of the EBMT. *Oncotarget* 2016; **7**: 43027–43038.

2 Baron F, Labopin M, Tischer J, Ciceri F, Raiola AM, Blaise D *et al.* Human leukocyte antigen-haploidentical transplantation for relapsed/refractory acute myeloid leukemia: Better leukemia-free survival with bone marrow than with peripheral blood stem cells in patients ≥55 years of age. *Am J Hematol* 2022; **97**: 1065–1074.

3 Glucksberg H, Storb R, Fefer A, Buckner CD, Neiman PE, Clift RA *et al.* Clinical manifestations of graft-versus-host disease in human recipients of marrow from HL-A-matched sibling donors. *Transplantation* 1974; **18**: 295–304.

4 Sorror ML, Maris MB, Storb R, Baron F, Sandmaier BM, Maloney DG *et al.* Hematopoietic cell transplantation (HCT)-specific comorbidity index: a new tool for risk assessment before allogeneic HCT. *Blood* 2005; **106**: 2912–2919.

5 Poiani M, Labopin M, Battipaglia G, Beelen DW, Tischer J, Finke J *et al.* The impact of cytogenetic risk on the outcomes of allogeneic hematopoietic cell transplantation in patients with relapsed/refractory acute myeloid leukemia: On behalf of the acute leukemia working party (ALWP) of the European group for blood and marrow transplantation (EBMT). *Am J Hematol* 2021; **96**: 40–50.

6 Nagler A, Labopin M, Canaani J, Niittyvuopio R, Socié G, Kröger N *et al.* Cytogenetic risk score maintains its prognostic significance in AML patients with detectable measurable residual disease undergoing transplantation in remission: On behalf of the acute leukemia working party of the European society for blood and marrow transplantation. *Am J Hematol* 2020. doi:10.1002/ajh.25905.

7 van Houwelingen HC. Dynamic prediction by landmarking in event history analysis. *Scand J Stat* 2007; **34**: 70–85.

8 van Houwelingen HC, Putter H. Dynamic predicting by landmarking as an alternative for multi-state modeling: an application to acute lymphoid leukemia data. *Lifetime Data Anal* 2008; **14**: 447–463.

9 Lin DY. The robust inference for the Cox proportional hazards model. *J Am Stat Assoc*; **84**: 1074–1078.

**Supplemental Table 1.** Patient characteristics

| **Population description** |  |  |
| --- | --- | --- |
| Follow-up (reverse Kaplan Meier) | median (95% CI) mo | 36.1 [32-44] |
| Status at transplant; # (%) | Primary refractory | 366 (54.6%) |
|  | Rel1 | 238 (35.5%) |
|  | Rel2+ | 66 (9.9%) |
| Cytogenetics; # (%) | Favorable | 27 (4%) |
|  | Intermediate | 336 (50.1%) |
|  | Adverse | 176 (26.3%) |
|  | NA/failed | 131 (19.6%) |
| Patient age (years) | median (min-max) [IQR] | 55.5 (18-78.8) [43-63] |
| Year transplant | median (min-max) | 2017 (2010-2020) |
| Diagnosis; # (%) | de novo AML | 543 (81%) |
|  | secondary AML | 127 (19%) |
| Cell source; # (%) | BM | 216 (32.2%) |
|  | PB | 454 (67.8%) |
| Karnofsky score; # (%) | <90 | 281 (44%) |
|  | >=90 | 357 (56%) |
|  | missing | 32 |
| HCT-CI; # (%) | HT-CI = 0 | 233 (48.4%) |
|  | HT-CI = 1 or 2 | 107 (22.2%) |
|  | HT-CI >=3 | 141 (29.3%) |
|  | missing | 189 |
| Previous auto HCT; # (%) | no | 650 (97%) |
|  | Yes | 20 (3%) |
| Patient sex; # (%) | Male | 383 (57.2%) |
|  | Female | 286 (42.8%) |
|  | missing | 1 |
| Donor sex; # (%) | donor male | 412 (61.7%) |
|  | donor female | 256 (38.3%) |
|  | missing | 2 |
| Female to male; # (%) | no F->M | 529 (79%) |
|  | F->M | 141 (21%) |
| Donor age (years) | median (min-max) [IQR] | 38.1 (13.1-73.9) [30-48] |
|  | missing (#) | 29 |
| Patient CMV; # (%) | Pat. CMV neg. | 159 (24.1%) |
|  | Pat. CMV pos | 502 (75.9%) |
|  | missing | 9 |
| Donor CMV; # (%) | Don. CMV neg. | 277 (42.2%) |
|  | Don. CMV pos | 379 (57.8%) |
|  | missing | 14 |
| Conditioning; # (%) | MAC | 289 (43.1%) |
|  | RIC | 381 (56.9%) |
| In vivo T-cell depletion; # (%) | no in vivo TCD | 585 (87.3%) |
|  | in vivo TCD | 85 (12.7%) |

mo, months; P refr, primary refractory; Rel1, first relapse; Rel2+, second or more advanced relapse; BM, bone marrow; PBSC, peripheral blood stem cells; HCT-CI, hematopoietic cell transplant-specific comorbidity index^4^; CMV, cytomegalovirus; MAC, myeloablative conditioning; RIC, reduced-intensity conditioning; F, female; M, male; in vivo TCT, in vivo T-cell depletion.

**Supplemental Table 2.** Final model stratified on landmark at time intervals from day of allo-HCT to day + 360 by 30 days excluding patients given *in vivo* T-cell depletion (i.e. ATG).

|  |  |  |  |  |  |  |  |  |
| --- | --- | --- | --- | --- | --- | --- | --- | --- |
|  | RI | | NRM | | LFS | | OS | |
|  | HR | p | HR | p | HR | p | HR | p |
| **acute GVHD II** | **0.94 (0.61-1.45)** | **0.78** | **0.91 (0.46-1.82)** | **0.8** | **0.94 (0.65-1.36)** | **0.75** | **1.03 (0.71-1.51)** | **0.86** |
| **acute GVHD III-IV** | **0.93 (0.56-1.55)** | **0.79** | **4.15 (2.52-6.83)** | **< 0.0001** | **1.64 (1.19-2.26)** | **0.002** | **1.58 (1.04-2.42)** | **0.033** |
| **limited cGVHD** | **0.79 (0.42-1.5)** | **0.48** | **1.14 (0.5-2.6)** | **0.75** | **0.9 (0.54-1.49)** | **0.67** | **0.81 (0.45-1.45)** | **0.48** |
| **extensive cGVHD** | **1.32 (0.7-2.47)** | **0.39** | **3.37 (1.87-6.08)** | **< 0.0001** | **1.95 (1.33-2.86)** | **0,0006** | **2.05 (1.36-3.09)** | **0.0006** |
| Age (per 10 y) | 0.9 (0.81-1) | 0.06 | 1.57 (1.27-1.94) | < 0.0001 | 1.04 (0.94-1.15) | 0.47 | 1.07 (0.96-1.19) | 0.2 |
| sec. AML | 0.75 (0.5-1.13) | 0.17 | 0.92 (0.55-1.53) | 0.75 | 0.83 (0.61-1.14) | 0.25 | 0.91 (0.67-1.26) | 0.58 |
| Adverse cytogenetics | 1.98 (1.45-2.71) | < 0.0001 | 1.4 (0.88-2.22) | 0.16 | 1.77 (1.37-2.28) | < 0.0001 | 1.73 (1.32-2.25) | < 0.0001 |
| Year of HSCT | 0.96 (0.91-1.02) | 0.18 | 0.99 (0.9-1.09) | 0.78 | 0.97 (0.92-1.02) | 0.28 | 0.98 (0.93-1.04) | 0.52 |
| KPS>=90 | 0.82 (0.62-1.1) | 0.19 | 0.57 (0.37-0.87) | 0.01 | 0.76 (0.59-0.96) | 0.023 | 0.76 (0.59-0.99) | 0.042 |
| female to male | 0.9 (0.62-1.3) | 0.57 | 1.2 (0.74-1.94) | 0.46 | 0.98 (0.74-1.3) | 0.9 | 1.11 (0.82-1.49) | 0.5 |
| Patient CMV positive | 1.2 (0.82-1.76) | 0.34 | 1.18 (0.7-2.01) | 0.53 | 1.18 (0.87-1.6) | 0.3 | 1.19 (0.87-1.64) | 0.27 |
| Donor CMV positive | 1.1 (0.81-1.5) | 0.55 | 0.79 (0.5-1.24) | 0.31 | 0.98 (0.76-1.27) | 0.91 | 0.99 (0.76-1.3) | 0.95 |
| PB vs BM | 0.94 (0.69-1.3) | 0.72 | 1.59 (0.97-2.59) | 0.064 | 1.07 (0.82-1.39) | 0.61 | 1.1 (0.84-1.45) | 0.49 |
| RIC vs MAC | 1.03 (0.75-1.41) | 0.86 | 0.9 (0.54-1.51) | 0.7 | 1.01 (0.77-1.33) | 0.92 | 1.13 (0.84-1.51) | 0.42 |

RI, incidence of relapse; NRM, nonrelapse mortality; LFS, leukemia-free survival; OS, overall survival; GVHD, graft-versus-host disease; cGVHD, chronic GVHD; HCT, hematopoietic cell transplantation; CMV, cytomegalovirus; PB, peripheral blood stem cells; BM, bone marrow; RIC, reduced-intensity conditioning; MAC, myeloablative conditioning; in vivo TCT, in vivo T-cell depletion.
